# Shift work and evening chronotype are associated with hepatic fat fraction and non-alcoholic fatty liver disease in 282,303 UK biobank participants

**DOI:** 10.1530/EC-23-0472

**Published:** 2024-01-12

**Authors:** Robert Maidstone, Martin K Rutter, Thomas Marjot, David W Ray, Matthew Baxter

**Affiliations:** 1 Oxford Centre for Diabetes, Endocrinology and Metabolism, and Oxford Kavli Centre for Nanoscience Discovery, University of Oxford, Oxford, UK; 2 Centre for Biological Timing, Faculty of Biology, Medicine and Health, University of Manchester, Manchester, UK; 3 Diabetes, Endocrinology and Metabolism Centre, Manchester University NHS Foundation Trust, NIHR Manchester Biomedical Research Centre, Manchester Academic Health Science Centre, Manchester, UK; 4 Oxford Liver Unit, Oxford University Hospitals NHS Foundation Trust, John Radcliffe Hospital, UK; 5 NIHR Oxford Health Biomedical Research Centre, and NIHR Oxford Biomedical Research Centre, John Radcliffe Hospital, Oxford, UK

**Keywords:** NAFLD, metabolism, shift work, circadian, chronotype

## Abstract

**Background and aims:**

Non-alcoholic fatty liver disease (NAFLD) has rapidly become the most common liver disease worldwide. Modern lifestyles have been linked to this rise in prevalence with changes in rhythmic human behaviour emerging as a possible mechanism. We investigated how shift working patterns and chronotype were associated with hepatic fat fraction and NAFLD in 282,303 UK Biobank participants.

**Methods:**

We stratified participants into day, irregular-shift, and permanent night-shift workers. We then utilised multiple methods of disease identification including (i) Dallas steatosis index (DSI), (ii) ICD10 codes, and (iii) hepatic proton density fat fraction (PDFF) and examined how shift work exposure impacted these variables. We further assessed the relationship of baseline chronotype with liver phenotypes using these same outcome measures.

**Results:**

Compared to day workers, irregular-shift workers were more likely to have a high DSI (OR 1.29 (1.2–1.4)) after adjusting for major covariates with some attenuation after additional adjustment for BMI (OR 1.12 (1.03–1.22)). Likelihood of high DSI was also increased in permanent night-shift workers (OR 1.08 (0.9–1.29)) in the fully adjusted model. Mediator analysis revealed that BMI was a significant mediator of the shift work effect. Compared to participants with intermediate chronotype, those with extreme late chronotype had a higher likelihood of high DSI defined NAFLD (OR 1.45 (1.34–1.56)) and a higher likelihood of NAFLD/NASH by ICD10 code (OR 1.23 (1.09–1.39)). Hepatic PDFF was elevated in irregular shift workers, but not permanent night-shift workers.

**Conclusions:**

Irregular-shift work and extreme late chronotype are associated with pathological liver fat accumulation, suggesting circadian misalignment may have an underlying pathogenic role. These findings have implications for health interventions to mitigate the detrimental effect of shift work.

## Introduction

Life in advanced societies is a challenge to evolutionarily conserved mechanisms regulating rhythmic human behaviour. In preindustrial societies sunlight exposure dominates organisation of activity, feeding, and fasting. The liver plays a key role in managing the predictable switching between the fed and fasted state, with 90% of lipogenic genes in the liver varying in expression through the day. Shift work is a highly prevalent challenge now, with nearly one in five exposed to shifts. This challenges the alignment between internal circadian time, and the external light–darkness environment, and potentially mediates some of the disease associations seen with shift work.

Pathological accumulation of fat in the liver drives non-alcoholic fatty liver disease (NAFLD). NAFLD seldom presents as a medical issue but can be detected on routine screening by detection of raised liver enzymes, or altered liver imaging parameters. NAFLD affects over 25% of the worldwide adult population, and the global prevalence has consistently increased over the last three decades (1, 2). Approximately 10–30% of people with NAFLD develop non-alcoholic steatohepatitis (NASH), which is associated with progression to liver fibrosis, cirrhosis, hepatocellular carcinoma, and cardiovascular disease (1). It is therefore of critical importance to establish the causes of this global epidemic of NAFLD/NASH.

Internal timing is driven by the core circadian clock which is sited in the hypothalamic suprachiasmatic nucleus (SCN). The SCN receives light information from the retina. The timekeeping mechanism is composed of a transcriptional–translational feedback loop, which has an intrinsic period of approximately 24 h. Circadian rhythms are critically important for healthy regulation of metabolism and endocrine functions (3). Environmental and behavioural changes such as artificial light, jet lag, and shift-work drive misalignment between the circadian clock phase and the external environment (4). The existing literature reveals conflicts regarding the relationship between shift work and NAFLD. A study of 6881 Chinese steel workers found that rotating night-shift work was associated with a higher likelihood of NAFLD as defined by ultrasound, with risks increasing with longer duration of shift work and longer night-time hours (5). Another study, incorporating 4740 male workers, found an association of night-shift work with elevated alanine transaminase (ALT) levels (6). However, the largest prior study included 8159 workers and showed no association between shift work and NAFLD as defined by liver enzyme levels (7). All of these studies have significant limitations, including relatively small sample sizes, lack of comparison between rotating and permanent night-shift workers, and use of imprecise methods for diagnosing NAFLD. Studies were also restricted to single occupations and racial groups which limit external generalisability.

Here, we aim to address the limitations of prior work by providing a comprehensive analysis of associations between several shift work subtypes and various parameters of liver fat accumulation, and where available estimations of NAFLD/NASH using a large and diverse UK cohort. Further, we test the potential mechanistic role of circadian disruption in NAFLD/NASH by examining the relationships with chronotype, the intrinsic preference for morning or evening activity.

## Methods

The UK Biobank recruited 502,540 participants, registered with the UK National Health Service (NHS) between 2007 and 2010. Participants were aged 40–69 years and completed baseline questionnaires on occupation, work hours, medical history, and lifestyle. Further information on medical conditions, medication and health status was gathered by trained healthcare professionals. Recruitment to the UK biobank was approved by North West Multi-centre Research Ethics Committee (MREC: 21/NW/0157) and was conducted in accordance with the Declaration of Helsinki. Consent has been obtained from each patient or subject after full explanation of the purpose and nature of all procedures used.

### Assessment of shift work

Analysis of shift work was performed on participants who were in paid employment or self-employed at time of baseline assessment (*n* = 286,825; Table 1). Participants were asked the following questions: Does your work involve shift work? The options presented were ‘Never/Rarely’, ‘Sometimes’, ‘Usually’, ‘Always’, ‘Do not know’, and ‘Prefer not to answer’. Those who answered ‘Do not know’ or ‘Prefer not to answer’ were excluded from further analysis. Those who answered ‘Never/Rarely’ were categorised as ‘Day workers’, and those who answered ‘Sometimes’, ‘Usually’, or ‘Always’ were subsequently asked: Does your work involve night shifts? The options presented were ‘Never/Rarely’, ‘Sometimes’, ‘Usually’, ‘Always’, ‘Do not know’, ‘Prefer not to answer’. Those who answered ‘Do not know’ or ‘Prefer not to answer’ were excluded. Those who answered ‘Never/Rarely’, ‘Sometimes’, or ‘Usually’ were categorised as ‘Irregular shift workers’, and those who answered ‘Always’ were categorised as ‘Permanent night workers’. Participants were thus categorised into three distinct groups: day workers (control), irregular shift workers, and permanent night workers.

**Table 1:** Participant characteristics by shift work status (*n* = 276,043).

	Current work schedule		
	Day workers	Irregular shift work	Permanent night-shift work
*n*	228,417	40,746	6880
Age (years)	53.34 (7.08)	52.36 (6.96)	51.95 (6.85)
Sex (% male)	46.01	53.39	61.1
BMI (kg/m^2^)	27.08 (4.65)	27.96 (4.96)	28.51 (4.89)
Smoker (%)			
Heavy smoker	6.32	10.79	13.44
Previously heavy smoker, occasional smoker now	0.95	1.17	1.06
Previously heavy smoker, not a smoker now	19.88	20.48	21.03
Occasional smoker	1.99	2.44	2.49
Previously an occasional smoker, not a smoker now	11.65	10.76	9
Never smoker	59.21	54.36	52.97
Smoking pack-years	5.49 (12.21)	7.7 (14.9)	9.22 (16.49)
Drink alcohol daily (%)	20.14	16.2	10.12
Monthly units of alcohol consumed^a^	56.26 (71.98)	57.14 (83.7)	50.87 (74.65)
Sleep duration (h)	7.05 (1.02)	6.92 (1.22)	6.69 (1.48)
Chronotype (%)			
Morning	23.37	24.5	19.39
Intermediate	58.76	56.04	49.65
Evening	7.97	8.66	16.87
Ethnicity (%)			
White British	88.58	82.14	81.21
White other	6.43	7.05	6.03
Mixed	0.65	0.93	0.84
Asian	1.68	3.56	3.33
Black	1.39	3.6	5.39
Chinese	0.34	0.46	0.67
Other	6.43	7.05	6.03
Weekly work hours	34.71 (12.58)	37.61 (12.91)	39.96 (13.19)
Single occupancy (%)	15.58	18.64	18.35
Townsend index	−2.24 (−3.7 to 0.18)	−1.31 (−3.2 to 1.68)	−1.04 (−3.03 to 2.06)
Maternal smoking (%)	26.7	29.27	31.02
Birth weight (kg)^a^	3.33 (0.63)	3.32 (0.68)	3.31 (0.71)
High cholesterol (%)	7.73	8.4	9.29
Type I diabetes (%)	0.07	0.05	0.06
Type II diabetes (%)	0.4	0.57	0.6
Hypertension (%)	20.17	22.08	23.1
Depression (%)	4.63	5.09	4.72
Cardiovascular disease (%)	3.72	4.24	4
Dallas steatosis index (DSI)	−2.65 (1.39)	−2.42 (1.42)	−2.26 (1.4)

Data are mean (s.d.) or percentages. DSI (Dallas steatosis index) is defined in Appendix A.

^a^Indicates variable with >20% missing data from participants.

### Diagnosis of NAFLD/NASH

We defined NAFLD and/or NASH using three methods: First, we used the Dallas steatosis index (DSI) (8) to define the probability of NAFLD at baseline, which is based on a logit prediction model incorporating age, diabetes, hypertension, triglyceride, and ethnicity variables (Appendix A, see the section on supplementary materials given at the end of this article). Second, we used prior hospital admissions associated with baseline ICD10 codes for either NAFLD and/or NASH. Finally, in an exploratory analysis, we used proton density fat fraction (PDFF) to define liver fat percentage in a sub-group of 6419 UK Biobank participants undergoing liver MR scanning (between August 2014 and October 2015). We used a 5.5% PDFF threshold to define NAFLD (9), and using this measure compared NAFLD prevalence at follow-up by baseline shift work status.

### Definition of chronotype

Chronotype was self-reported at baseline by answering a questionnaire. Participants were asked whether they consider themselves to be: ‘Definitely a “morning” person’, or ‘More a “morning” than an “evening” person’, or ‘More an “evening” than a “morning” person’, or ‘Definitely an “evening” person’, or ‘Don’t know’, or ‘Prefer not to answer’. We combined people responding, ‘More a “morning” than an “evening” person’ and ‘More an “evening” than a “morning” person’ to form referent group against which we compared outcomes in subjects responding, ‘Definitely a “morning” person’ or ‘Definitely an “evening” person’. Participants answering ‘Do not know’ or ‘Prefer not to answer’ were excluded. Self-reported chronotype defined in this way has been shown to correlate with dim-light melatonin onset and sleep timing (10, 11, 12).

### Statistical analysis

Multivariate logistic regression models compared adjusted odds ratios and corresponding 95% asymptotic confidence intervals for NAFLD outcomes in different exposure groups, with day workers and people with intermediate chronotype serving as referent groups. Model 1 was adjusted for sex, age, ethnicity, and Townsend deprivation index (TDI). Model 2 adjusted for model 1 covariates plus sleep duration (categorised as short sleepers; <6 h, normal sleepers; and long sleepers, >8 h) alcohol intake frequency (‘daily or almost daily’, ‘three or four times a week’, ‘once or twice a week’, ‘one to three times a month’, ‘special occasions only’ or ‘never’), smoking status (current, previous, or never), smoking pack-years (number of cigarettes smoked per day, divided by twenty, multiplied by the number of years of smoking), and length of working week. Finally, model 3 contained model 2 covariates plus BMI, which could be a mediator and/or potential confounder for the association between shift work and NAFLD/NASH. Participants with missing data for covariates were excluded.

## Results

Out of 286,825 UK Biobank participants studied, 42,786 were irregular shift workers and 7142 were permanent night-shift workers, the remainder being day workers (Table 1). Irregular and permanent night-shift workers were older, more likely to be male and of non-White ethnicity and had higher BMI values compared to day workers. They were also more likely to smoke but less likely to drink alcohol compared to day workers.

### Irregular and permanent night-shift work is associated with greater predicted pathological liver fat content

**Figure 1:**
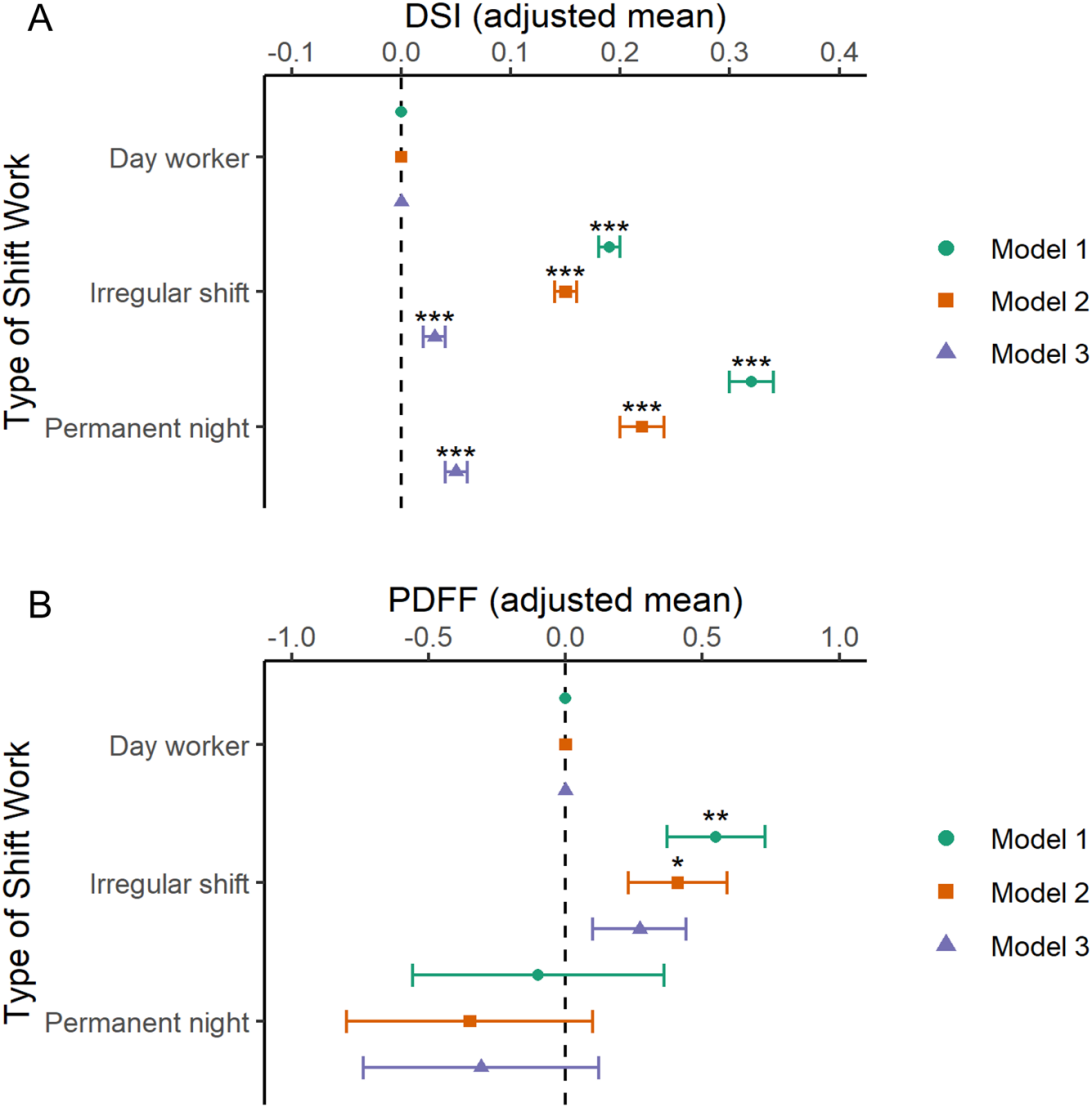
Shift work is associated with NAFLD. Participants were categorised as day workers, irregular shift workers, or permanent night-shift workers. (A) Adjusted mean (95% CI) of Dallas steatosis index (DSI), *n* = 236,089. (B) Adjusted mean (95% CI) of liver fat as measured by proton density fat fraction (PDFF), *n* = 6324. Model 1 was adjusted for sex, age, ethnicity, and Townsend deprivation index (TDI). Model 2 adjusted for model 1 covariates plus sleep duration alcohol intake frequency, smoking status, smoking pack-years, and length of working week. Model 3 contained model 2 covariates plus BMI. Statistical significance was determined by Student’s *t*-test. *P < 0.05; **P < 0.01; ***P < 0.001.

A problem we encountered in the UK Biobank data was the very low prevalence of diagnosed NAFLD. This reflects the difficulty in detecting cases of a disease state which is essentially silent. However, we determined to use the the DSI as an approach to estimate the prevalence of NAFLD. The DSI takes a number of frequently measured parameters to estimate the likelihood of NAFLD. DSI categorises the risk of NAFLD as low (<20% predicted risk), intermediate (20–49% risk), or high (≥50% risk). The DSI generates a value between −2.2 (= 10% probability of NAFLD) and >2.2 (= 90% probability of NAFLD). The threshold for 50% probability of NAFLD is a score of 0. Here, we use DSI score both as a continuous variable (Fig. 1A and Supplementary Table 1B), and have also explored a conservative estimate for prediction of NAFLD of 60% for a categorical comparison (Supplementary Table 1A). We compared the different work schedule populations.

Out of 240,589 UK Biobank participants, mean DSI scores were significantly higher in both irregular shift workers and in permanent night-shift workers compared to day workers in all three models (Fig. 1A and Supplementary Table 1B). Differences in DSI values when comparing shift workers with day workers were markedly attenuated, but remained significant, after adjusting for BMI (model 3; Fig. 1A and Supplementary Table 1B). When defining risk of NAFLD using DSI score (>60%), higher odds of NAFLD was observed in the permanent night-shift workers compared to day workers in models 1 and 2, but not after further adjustment for BMI in model 3 (Supplementary Table 1A). Using this method, we found a higher likelihood of NAFLD in irregular shift workers in all 3 models.

### Shift work is not significantly linked to NAFLD/NASH by ICD10 code, but this may be explained by under-diagnosis

We examined the recorded disease codes relevant for NAFLD and NASH, although we anticipated a high proportion of missing cases, especially in the NAFLD group. We did not identify a higher likelihood of NAFLD/NASH in shift workers when the diagnosis was based on baseline ICD 10 coding (Supplementary Table 2). However, the odds ratios had wide confidence intervals suggesting that this analysis was underpowered. It is recognised that most NAFLD/NASH remains undiagnosed and is missed by ICD10 coding. We observed this in the UK Biobank, where in day workers, irregular shift workers, and permanent night workers, the prevalence of NAFLD or NASH by ICD10 diagnosis of was 0.08, 0.1, and 0.11%, respectively (Supplementary Table 2). NAFLD is thought to have a prevalence >25% in UK. ICD10 code diagnosis is therefore an inappropriate approach to examine pathological liver fat accumulation in biobank populations.

### Irregular shift work is associated with high liver fat fraction

We next conducted an exploratory analysis of the association between shift work and liver fat, as measured by proton density fat fraction (PDFF). PDFF is a direct measure of liver fat. This is only available in a subset of UK Biobank participants, and although inclusion was determined by enrolment centre (some were designated imaging centres and some not) there remains potential bias in selection. We analysed liver fat as a continuous variable in different work schedule populations (Fig. 1B and Supplementary Table 3B), and secondly as a categorical variable using >5.5% fat as a cut-off for the definition of non-alcoholic fatty liver (Supplementary Table 3A) (9). When compared to day shift workers, irregular shift workers, but not permanent night-shift workers, had a higher mean PDFF (Fig. 1B, model 1) values and a higher odds of elevated liver fat (>5.5%) (Supplementary Table 3A; model 1). Attenuation of these relationships were observed in models 2 and 3 when the elevated liver fat outcome was considered as a binary outcome variable. However, when PDFF was considered as a continuous variable, irregular shift workers had higher levels of liver fat when compared to day workers in models 1 and 2, although statistical significance was lost after further adjustment for BMI in model 3 (Fig. 1B).

The attenuation of the shift work effect when controlled for BMI raised the possibility that BMI or obesity may be a mediator of the shift work effect. To examine this we conducted formal mediator analysis, using a bootstrapping approach (13) via the r package ‘mediation’. This did indeed reveal highly significant mediator effect, *P* < 0.01. This also estimates that of the shift work/NAFLD effect approximately 0.36 (36%) is being mediated by BMI. BMI, and obesity are recognised to be increased in shift workers.

### Extreme chronotype is associated with greater risk of NALFD/NASH

**Figure 2:**
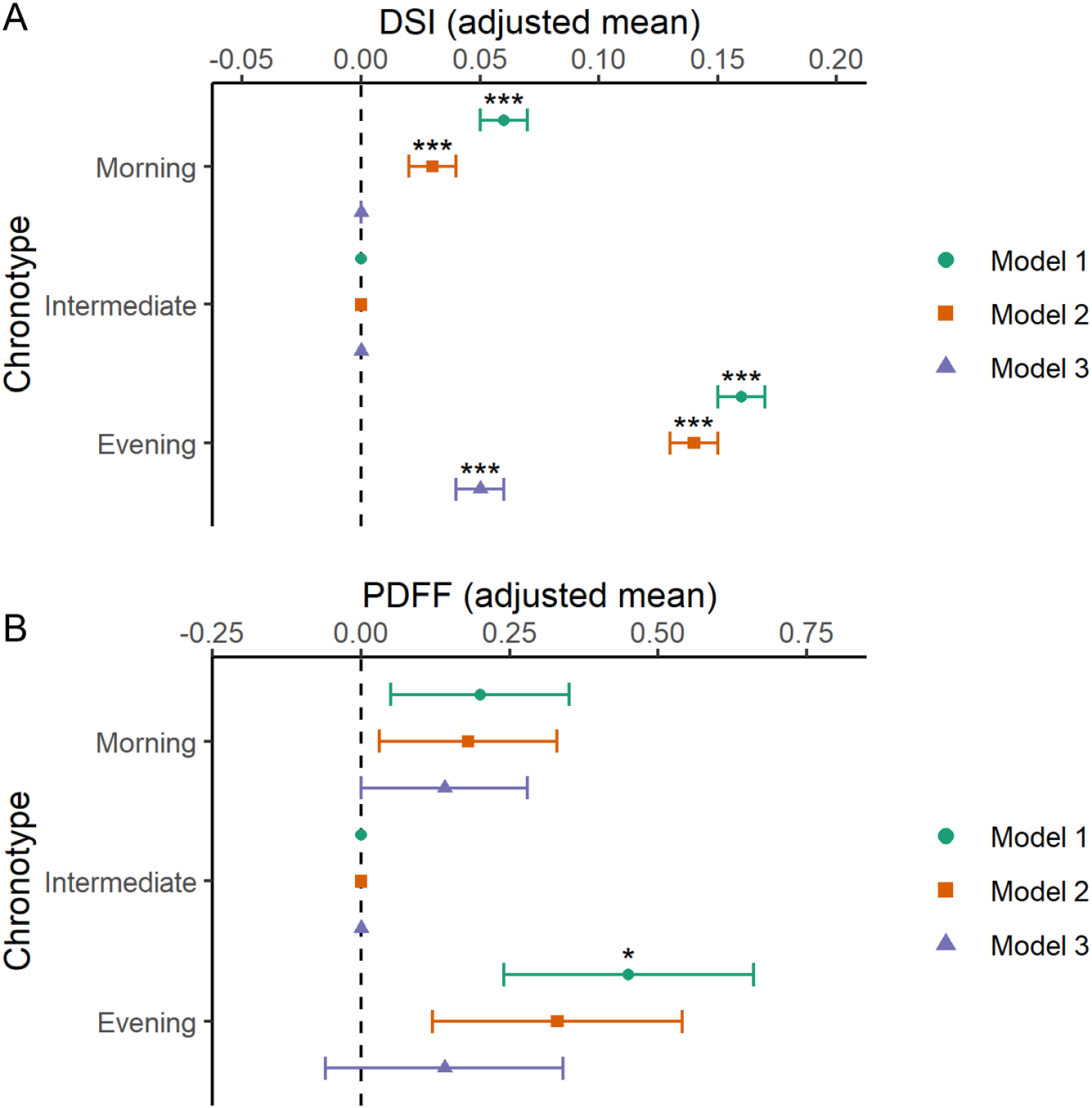
Extreme chronotype is associated with NAFLD. Participants were categorised as extreme morningness (’morning’), extreme eveningness (‘evening’) and compared to control ‘intermediate’ chronotype. (A) Adjusted mean of Dallas steatosis index (DSI), *n* = 368,612. (B) Adjusted mean of liver fat as measured by proton density fat fraction (PDFF), *n* = 8490. Model 1 was adjusted for sex, age, ethnicity and Townsend deprivation index (TDI). Model 2 adjusted for model 1 covariates plus sleep duration alcohol intake frequency, smoking status, smoking pack-years, and length of working week. Model 3 contained model 2 covariates plus BMI. Statistical significance was determined by Student’s *t*-test. *P < 0.05; **P < 0.01; ***P < 0.001.

We hypothesised that the increased risk of pathological liver fat accumulation in certain groups of shift workers may be due to circadian misalignment. In order to test this hypothesis we examined the relationship between chronotype and liver fat parameters. Individuals with extreme chronotypes may experience circadian misalignment in a similar way to shift workers (14, 15). Compared to individuals with an intermediate chronotype, the mean DSI values were significantly higher in the strong eveningness group in all 3 models, although adjustment for BMI (model 3) attenuated the relationship (Fig. 2A and Supplementary Table 4A). Mean DSI values were also higher in the strong morningness group in models 1 and 2, although this was entirely abrogated in model 3. There was a significant association between strong eveningness and having a high probability of NAFLD (DSI >60%) and this persisted in all models (Supplementary Tables 4A and B). In the exploratory analysis, neither strong eveningness, nor strong morningness was significantly associated with high PDFF (>5.5%; Supplementary Table 6A). However, average PDFF was higher in the strong eveningness group than the intermediate group in the minimally adjusted model (model 1; Fig. 2B). To examine whether the association between shift work and liver disease was influenced by chronotype, we stratified the participants into three subsets; definite morning, definite evening, and intermediate chronotypes. In all chronotype subgroups there was a significant association between shift work and DSI. However, a significant interaction by chronotype status (*P*_interaction_ < 0.01) suggested that the relationship between shift work and DSI was stronger in morning than in evening chronotypes (Supplementary Table 7).

## Discussion

In this study we show that shift workers have higher odds of pathological liver fat accumulation than non-shift workers. The way in which these relationships were attenuated on adjustment for BMI are in keeping with a mediating role of obesity, which was more prevalent in shift workers when compared to day workers. Furthermore, extreme late chronotype was also associated with increased odds of accumulating liver fat content, and predisposing to various diseases including NAFLD. Individuals with an extreme late chronotype exist in a state of circadian misalignment between their internal circadian clock, and the environmental light–darkness cycle, and behavioural activity–feeding–rest cycle. We also find that there is an interaction between chronotype, and shift work exposure when it comes to liver fat content, suggesting that those with morning chronotype may be at greatest risk of developing metabolic liver disease as a consequence of shift work. Taken together, these observations provide evidence that circadian misalignment resulting from shift work exposure may contribute to pathological liver fat accumulation, and to its associated diseases.

Our findings have immediate and clear clinical and public health implications. Liver fat accumulation lies at the heart of prevalent human diseases including NAFLD, and presents as an increasingly important global health burden. Cases of NAFLD worldwide have risen from 391.2 million in 1990 to 882.1 million in 2017 (16). Recent modelling data has forecast that prevalent NAFLD cases will increase 21% between 2015 and 2030, and prevalent NASH cases, which emerge from NAFLD, will increase by 63%. In real terms this projection means a NAFLD prevalence in the adult population of 33.5% by 2030. In the same period, the proportion of NAFLD cases classified as NASH will increase from 20% to 27%. Liver deaths worldwide will increase by 178% (17). These stark figures underline a burgeoning health crisis, and a worldwide unmet clinical need. Much focus has been drawn to the expansion of ‘western lifestyles’ in terms of diet and behaviour as an explanation for this epidemic. In particular, the increasing prevalence of circadian disruptive work patterns and social jet lag, caused by shift work, parallels the increasing prevalence of NAFLD and NASH. To date, most research into circadian disruption and the development of NAFLD/NASH has employed mouse models (18, 19), and evidence in humans has been conflicting and limited by small sample sizes (5, 6, 7). Here, we present the first evidence associating disrupted circadian schedules to NAFLD and NASH in humans. However, it should be noted that interpreting shift work and chronotype data is complex, as related factors such as sleep quality, social jet lag, dietary patterns, and dietary quality are often reported as key factors in determining adverse health associations.

Our findings contradict those of Balakrishnan *et al*.,and broadly align with those of Zhang *et al.* (5, 7). Our study has the clear advantage of scale, allowing us to identify associations with far greater confidence, and to consider a range of possible mediators and confounders. Given our large cohort size we were also able to stratify shift workers by whether they work irregular shift patterns or whether they work permanent nights. Interestingly, Zhang *et al.* found a positive association between the duration of night shifts and NAFLD as well as with the cumulative length of night shifts and NAFLD. We considered various approaches to detect cases of NAFLD. Here, we used DSI score as a robust measure which we could apply across the UK Biobank population. DSI score is not the same as a formal diagnosis of NAFLD, but as the recorded ICD10 codes had only detected a tiny proportion of the predicted NAFLD patients the DSI score was preferred. The DSI also had the advantage that all the data were collected at the time of enrolment, when work schedule was captured. DSI has previously been applied to estimate NAFLD in biobank studies, including the UK Biobank (8). We found an association between both irregular and permanent night-shift work and the mean DSI score. Interestingly, the strength of relationships were markedly reduced when BMI was included in the models as a covariate. These data are consistent with BMI being a confounder and/or a mediator of the relationship between shift work and NAFLD; our prior work has shown that obesity is associated with selection into shift work (20) and other work suggests that obesity may be a consequence of shift work (21).

It is possible that permanent night shifts may allow closer alignment of the internal circadian clock phase with the external environmental, and the behavioural cycles. This finding led us to test the role of circadian misalignment in a new analysis on extreme chronotypes, and indeed we discovered an association with extreme late chronotypes, when using the DSI as the diagnostic criterion. This is a striking finding, which led us to test for an interaction between night-shift work, chronotype, and DSI, which suggested that people with morning chronotypes may be at especially high risk of developing NASH in association with shift work. This intriguing hypothesis will need to be confirmed in prospective studies. Establishing an interaction between these three parameters could have important implications for health screening of shift workers, and could point to strategies to reduce the risk associated with night shifts, such as recommended eating schedules. Interestingly, in earlier work we also discovered a relationship between early chronotypes and risk of asthma liked to shift work (14).

Our results should be interpreted within the context of several study limitations. PDFF measurements were only available in a small proportion of the participants (6482 workers), which resulted in large confidence intervals. Furthermore, the PDFF measurements were not conducted at the same time as work pattern assessments and did not account for differential deaths rates by shift work status during follow-up. We were also unable to examine the role of shift work in the progression of NAFLD to NASH, due to difficulties in making confident diagnoses in the UK Biobank population.

Understanding the role of circadian biology in human populations is critically important to move the advances in the field into the clinic. Liver is a highly rhythmic organ, and subject to highly rhythmic inputs in terms of food timing. Indeed, the phase of the liver clock will follow food timing, shifting by about an hour per day. Fat metabolism in the liver is highly rhythmic, and so investigating how liver fat metabolism is perturbed by shift work is an obvious and essential step. In addition, drugs targeting the liver may have differential effects depending on the time of day of administration. For example, statin drugs work best at night, and therapeutic glucocorticoids show differential effects depending on circadian phase, due to circadian modulation of glucocorticoid receptor cistromes (22).

## Conclusion

This study shows that shift workers have a higher likelihood of developing pathological liver fat content, predisposing them to related diseases such as NAFLD and NASH. This progression is likely mediated by circadian misalignment, as those with extreme chronotypes share a similar liver fat accumulation. We have discovered that the shift work effect is mediated by obesity, which identifies a way in which the risks of shift work could be mitigated, and an approach that could protect shift workers from metabolic harms. Our modelling data also suggest interaction effects so that individuals with morning chronotype may be at greatest risk of the metabolic harms of shift work. Understanding the mechanisms linking shift work to pathogenic liver fat accumulation may identify further therapeutic targets or modifying behaviours for shift workers to reduce the disease risk.

## Supplementary materials

Supplementary Material

## Declaration of interest

The authors declare no conflicts of interest.

## Funding

The work is supported by an MRC programme grant MR/P023576/1. DWR is supported by NIHR Oxford Health Biomedical Research Centre grant reference number: NIHR203316. MRC MR/W019000/1 and MR/V034049/1. TM is funded via a Wellcome Trusthttp://dx.doi.org/10.13039/100010269 Clinical Research Training Fellowship (ref. 102176/B/13/Z).

## Author contribution statement

All authors contributed to the design of the study, interpretation of the data and writing of the manuscript.
