# Supplementary material for: Shift work and evening chronotype are associated with hepatic fat fraction and non-alcoholic fatty liver disease in 282,303 UK biobank participants
Source: Endocr Connect. 2024 Jan 12;13(2):e230472. doi: 10.1530/EC-23-0472 (PMC10831536; doi:10.1530/EC-23-0472)
Supplement: Supplementary Material [file supplementary_material.pdf]

Supplemental Table 1a: Adjusted odds (95% CI) of having high probability of NAFLD (p> 0.6, calculated from the DSI) by shift work schedule (N = 236,089)

	Current work schedule		
	Day workers	Irregular shift work	Always
Total cases (% of total sample size)	2,747 (1.41%)	733 (2.1%)	138 (2.34%)
Total sample size	195,218	34,976	5,895
Model 1: Age, Sex, Ethnicity and TDI adjusted.	1	1.42 (1.3-1.54)	1.57 (1.32-1.87)
Model 2: Model 1 covariates + Sleep Duration, Alcohol, Smoking and Length of working week.	1	1.28 (1.18-1.39)	1.26 (1.06-1.5)
Model 3: Model 2 covariates + BMI	1	1.11 (1.01-1.21)	1.08 (0.9-1.29)

Supplemental Table 1b: Adjusted means (standard error) of DSI by shift work schedule (N = 236,089)

	Current work schedule		
	Day workers	Irregular shift work	Always
Total sample size	195,218	34,976	5,895
Model 1: Age, Sex, Ethnicity and TDI adjusted.	0	0.19 (0.01)***	0.32 (0.02)***
Model 2: Model 1 covariates + Sleep Duration, Alcohol, Smoking and Length of working week.	0	0.15 (0.01)***	0.22 (0.02)***
Model 3: Model 2 covariates + BMI	0	0.03 (0.01)***	0.05 (0.01)***

Statistical significance using Student's t-test. * indicates p<0.05, ** p<0.01 and *** p<0.001.

Supplemental Table 2: Adjusted odds (95% CI) of NAFLD or NASH by shift work schedule (N = 276,043)

	Current work schedule		
	Day workers	Irregular shift work	Always
Total cases (% of total sample size)	1,007 (0.44%)	261 (0.64%)	37 (0.54%)
Total sample size	228,417	40,746	6,880
Model 1: Age, Sex, Ethnicity and TDI adjusted.	1	1.37 (1.19-1.57)	1.13 (0.81-1.57)
Model 2: Model 1 covariates + Sleep Duration, Alcohol, Smoking and Length of working week.	1	1.25 (1.08-1.44)	0.93 (0.67-1.3)
Model 3: Model 2 covariates + BMI	1	1.15 (1-1.33)	0.85 (0.61-1.18)

Supplemental Table 3a: Adjusted odds (95% CI) of high proton density fat fraction (> 5.5%) by shift work schedule (N = 6,324)

	Current work schedule		
	Day workers	Irregular shift work	Always
Total cases (% of total sample size)	1,123 (20.59%)	190 (24.9%)	23 (21.3%)
Total sample size	5,453	763	108
Model 1: Age, Sex, Ethnicity and TDI adjusted.	1	1.26 (1.05-1.51)	0.98 (0.61-1.57)
Model 2: Model 1 covariates + Sleep Duration, Alcohol, Smoking and Length of working week.	1	1.17 (0.97-1.41)	0.87 (0.54-1.4)
Model 3: Model 2 covariates + BMI	1	1.1 (0.91-1.34)	0.87 (0.53-1.43)

Supplemental Table 3b: Adjusted means (standard error) of proton density fat fraction by shift work schedule (N = 6,324)

	Current work schedule		
	Day workers	Irregular shift work	Always
Total sample size	5,453	763	108
Model 1: Age, Sex, Ethnicity and TDI adjusted.	0	0.55 (0.18)**	-0.1 (0.46)
Model 2: Model 1 covariates + Sleep Duration, Alcohol, Smoking and Length of working week.	0	0.41 (0.18)*	-0.35 (0.45)
Model 3: Model 2 covariates + BMI	0	0.27 (0.17)	-0.31 (0.43)

Statistical significance using Student's t-test. * indicates p<0.05, ** p<0.01 and *** p<0.001.

Supplemental Table 4a: Adjusted odds (95% CI) of having high probability of NAFLD (p> 0.6, calculated from the DSI) by chronotype (N = 368,612)

	Chronotype		
	Intermediate chronotype	Definitely a morning person	Definitely an evening person
Total cases (% of total sample size)	4,036 (1.71%)	1,954 (1.96%)	890 (2.7%)
Total sample size	235,779	99,884	32,949
Model 1: Age, Sex, Ethnicity and TDI adjusted.	1	1.09 (1.04-1.16)	1.55 (1.44-1.67)
Model 2: Model 1 covariates + Sleep Duration, Alcohol, Smoking and Length of working week.	1	1.03 (0.98-1.09)	1.45 (1.34-1.56)
Model 3: Model 2 covariates + BMI	1	1 (0.94-1.06)	1.29 (1.19-1.39)

Supplemental Table 4b: Adjusted means (standard error) of DSI by chronotype (N =368,612)

	Chronotype		
	Intermediate chronotype	Definitely a morning person	Definitely an evening person
Total sample size	235,779	99,884	32,949
Model 1: Age, Sex, Ethnicity and TDI adjusted.	0	0.06 (0.01)***	0.16 (0.01)***
Model 2: Model 1 covariates + Sleep Duration, Alcohol, Smoking and Length of working week.	0	0.03 (0.01)***	0.14 (0.01)***
Model 3: Model 2 covariates + BMI	0	0 (0)	0.05 (0.01)***

Statistical significance using Student's t-test. * indicates p<0.05, ** p<0.01 and *** p<0.001.

Supplemental Table 5: Adjusted odds (95% CI) of NAFLD or NASH by chronotype (N = 427,678)

	Chronotype		
	Intermediate chronotype	Definitely a morning person	Definitely an evening person
Total cases (% of total sample size)	1,529 (0.56%)	689 (0.59%)	302 (0.79%)
Total sample size	273,479	115,997	38,202
Model 1: Age, Sex, Ethnicity and TDI adjusted.	1	1.01 (0.92-1.11)	1.36 (1.2-1.54)
Model 2: Model 1 covariates + Sleep Duration, Alcohol, Smoking and Length of working week.	1	0.97 (0.89-1.06)	1.24 (1.09-1.4)
Model 3: Model 2 covariates + BMI	1	0.96 (0.87-1.05)	1.16 (1.02-1.32)

Supplemental Table 6a: Adjusted odds (95% CI) of high proton density fat fraction (> 5.5%) by chronotype (N = 8,490)

	Chronotype		
	Intermediate chronotype	Definitely a morning person	Definitely an evening person
Total cases (% of total sample size)	1,149 (20.4%)	443 (21.77%)	194 (23.57%)
Total sample size	5,632	2,035	823
Model 1: Age, Sex, Ethnicity and TDI adjusted.	1	1.08 (0.96-1.23)	1.17 (0.98-1.39)
Model 2: Model 1 covariates + Sleep Duration, Alcohol, Smoking and Length of working week.	1	1.07 (0.94-1.21)	1.1 (0.92-1.31)
Model 3: Model 2 covariates + BMI	1	1.04 (0.91-1.19)	1 (0.82-1.2)

Supplemental Table 6b: Adjusted means (standard error) of proton density fat fraction by chronotype (N = 8,490)

	Chronotype		
	Intermediate chronotype	Definitely a morning person	Definitely an evening person
Total sample size	5,632	2,035	823
Model 1: Age, Sex, Ethnicity and TDI adjusted.	0	0.2 (0.15)	0.45 (0.21)*
Model 2: Model 1 covariates + Sleep Duration, Alcohol, Smoking and Length of working week.	0	0.18 (0.15)	0.33 (0.21)
Model 3: Model 2 covariates + BMI	0	0.14 (0.14)	0.14 (0.2)

Statistical significance using Student’s t-test. * indicates p<0.05, ** p<0.01 and *** p<0.001.

Supplemental Table 7: Adjusted means (standard deviation) of DSI in night shift workers when compared to day workers, stratified by chronotype

Current work schedule	Mean (standard deviation)	$P_{interaction}$
**Definite morning chronotype (N= 55,384)**		
Day workers	ref	<0.01
Irregular shift work including nights	0.14 (0.02)***	
Permanent night shift work	0.26 (0.04)***	
**Intermediate chronotype (N= 137,190)**		
Day workers	ref	
Irregular shift work including nights	0.15 (0.01)***	
Permanent night shift work	0.20 (0.02)***	
**Definite evening chronotype (N= 19,595)**		
Day workers	ref	
Irregular shift work including nights	0.10 (0.03)***	
Permanent night shift work	0.13 (0.05)**	

Statistical significance of adjusted means using Student's t-test. * indicates p<0.05, ** p<0.01, and *** p<0.001. Interaction analysis; likelihood ratio test comparing models with/without an interaction term.

Appendix A

The Dallas Steatosis Index (DSI) was defined as a LOGIT equation as derived in McHenry et al (2020):

$$
\begin{aligned}
DSI^{LOGIT} = \; & -9.4 + 0.316(if\ age \geq 50\ \&\ female) + 2.4(if\ any\ diabetes) \\
+ \quad & 0.02(if\ no\ diabetes)(Glucose\ concentration\ in\ mg/dL) \\
+ \quad & 0.3(if\ hypertension) \\
+ \quad & 0.5(Ethnicity\ is\ Asian, Chinese\ or\ Other) \\
+ \quad & \log(Triglycerides\ in\ mg/dL) + 0.4(13.5 \leq ALT < 19.5) \\
+ \quad & 1.1(19.5 \leq ALT \leq 40) + 1.5(ALT > 40) \\
+ \quad & 0.7\big(Ethnicity\ is\ not\ black\ \&\ (25 \leq BMI < 27.5)\big) \\
+ \quad & 1.4\big(Ethnicity\ is\ not\ black\ \&\ (27.5 \leq BMI < 35)\big) \\
+ \quad & 1.9\big(Ethnicity\ is\ not\ black\ \&\ (35 \leq BMI < 37.5)\big) \\
+ \quad & 2.6(Ethnicity\ is\ not\ black\ \&\ BMI > 37.5) \\
+ \quad & (-0.2)\big(Ethnicity\ is\ black\ \&\ (25 \leq BMI < 27.5)\big) \\
+ \quad & 0.8\big(Ethnicity\ is\ black\ \&\ (27.5 \leq BMI < 35)\big) \\
+ \quad & 0.8\big(Ethnicity\ is\ black\ \&\ (35 \leq BMI < 37.5)\big) \\
+ \quad & 1.8(Ethnicity\ is\ black\ \&\ BMI > 37.5)
\end{aligned}
$$

We defined the probability of NAFLD using the following formula and defined high-risk as a probability >0.6:

$$P(NAFLD) = \frac{\exp(DSI)}{1 + \exp(DSI)}$$
